# Two Cases of Stone in the Bladder

**Published:** 1855-05

**Authors:** William H. Gardner

**Affiliations:** Woodsonville, Ky.


*December*, 1854.

### ART. III.—TWO CASES OF STONE IN THE BLADDER. LITHOTOMY.

BY WILLIAM H. GARDNER, M. D., WOODSONVILLE, KY.

CASE 1. Samuel Shitton Clemmons, aged four and a half years. Oct. 22, 1853. Was a tolerably healthy child during his first year; at that age his mother had a long and severe spell of illness while he yet sucked. He became sickly and suffered much during his second summer with chronic diarrhœa, from which he recovered slowly during the fall and winter. About this time he began to suffer pain in passing water, which has continued up to the present time, now two and a half years. On sounding him to day a calculus of considerable size was found.

Oct. 24. I proceeded to remove the stone, assisted by Drs. JETT, H. DAWSON, and Mr. HAMMON and Mr. BUMGARDNER, students of medicine, and Mr. W. L. KIDDER, chief engineer of Green River division of the Louisville and Nashville railroad. He was placed on the table at 12 o'clock; chloroform was administered by Dr. DAWSON, and the staff was given to Dr. JETT. I made the lateral operation in the usual manner, only I used the straight, probe-pointed bistoury in making the last incision in the neck of the bladder. The operation was completed without accident, difficulty, or delay, and the stone removed while the little patient was wholly unconscious. The stone was one inch and a quarter long, one inch wide by half an inch thick; composed chiefly of urate and phosphate of ammonia. During the evening he complained of smarting of the wound from urine passing; no hemorrhage.

25. Passed the night comfortably, only complained of some smarting of the wound. Directed him to keep quiet; no medicine whatever given, nor had he taken a grain by way of preparation before the operation.

26. Some fever; pulse accelerated, otherwise easy; urine by the natural channel.

27. Still doing well; urine all by the wound; dose of oil; bowels acted.

28. Patient doing well.

31. Patient still doing well; bowels rather active; opiate to check; urine all by the wound; no fever; appetite good; child cheerful and playful.

Nov. 6. Fourteenth day from the operation; wound healed; urine all by the natural course since day before yesterday; bowels have been rather active; opiate accordingly.

This patient was the son of a farmer who resided in Edmondson county, Ky., three miles east of the Mammoth Cave, on the elevated plateau or level of the country on which the Cave-House stands, which is, as estimated by Mr. KIDDER, about one hundred and fifty feet above the limestone. His father has never been below the sand-stone formation and has used "freestone" water altogether. This stone is in some respects peculiar, and does not resemble any calculus in my collection of some twenty-six, all extracted from patients using limestone water.

---

CASE 2. Mary, a slave the property of the estate of the late Dr. LEWIS BARRET, of Munfordsville, Ky., aged about fifty years, born in Halifax county, Virginia; has resided in Kentucky the last thirty years; the mother of ten children, the youngest now thirteen years of age. She has usually enjoyed good health until the last few years. Some seven years since she began to experience difficulty in urinating; has been growing much worse for the last three years, most of which time she passed in the city of Louisville. Has voided several small calculi, four of which she gave me; the largest is half an inch in diameter, oval, flat, and very hard, it was passed off some twelve months ago. She now has frequent calls to pass water, attended with severe pain, straining and vesical tenesmus. Sounding detected at once the presence of a large stone.

June 3, 1854. Having derived little or no benefit from a few day's preparatory treatment, I determined to remove the stone as the patient's sufferings were very great. Assisted by Dr. JETT, Dr. BROWN, Dr. DAWSON, and Mr. BUMGARDNER, I proceeded to operate in the following manner:—I introduced a grooved director into the urethra and with the probe-pointed bistoury made an incision from below upwards, in the direction of the symphisis pubis, dividing the urethra in about half its extent. This admitted the forefinger at once into the bladder with which I dilated and searched the bladder, finding the stone, or rather stones, for there were two of them, large, I discovered that to remove them by dilating alone was out of the question. I therefore passed the bistoury, with the flat side on my finger, and incised both sides of the neck of the bladder, as practised by Mr. LISTON. This was sufficient to admit two fingers, but did not afford room enough yet to allow the stone to pass. I next divided the urethra downwards into the vagina extending the incision about two-thirds the extent of the urethra. The forceps were then introduced and the small stone extracted, after which the larger one was removed with more difficulty. The two calculi weighed six ounces and a quarter. The patient was under the influence of chloroform administered by my friend, Dr. DAWSON, and although the operation was one of much severity she was wholly unconscious of what was going on. The bladder was examined and washed out with warm water, and the patient removed from the table, when she slept comfortably, perhaps an hour, after which she complained of smarting of the wound. An opiate was given.

4. Patient rested well last night; no fever or excitement.

5. Still doing well; patient altogether comfortable.

7. Patient doing well; appetite good; no action of the bowels since the operation; dose of oil to night; urine expelled involuntarily at intervals of two hours.

11. Patient doing well; appetite good; oil again to-day which acted well.

17. Patient wishes to be up, I do not allow her yet to rise; bowels acted last night without medicine, third action since the operation; she is able to retain the urine much longer; wound nearly healed.

24. Twenty-second day; wound all healed to small fissure at the orifice of the urethra; can retain the urine longer and rise to pass it off.

July 1. Patient well; up at work; retains her urine very well; when the bladder fills it becomes impatient to expel its contents, but gives her time to go aside.

